# *Atractylodes macrocephala* Koidzumi modulates human colonic motility via ICCs pacemaker suppression and cAMP/ATP-sensitive K⁺ channel pathways

**DOI:** 10.7150/ijms.116169

**Published:** 2025-07-24

**Authors:** Tae Sik Sung, Seok Jae Ko, Woo-Gyun Choi, Jae-Woo Park, Byung Joo Kim

**Affiliations:** 1 Department of Physiology, Kosin University College of Medicine, Busan 49267, Republic of Korea.; 2 Department of Clinical Korean Medicine, Graduate School of Kyung Hee University, Seoul 02447, Republic of Korea.; 3 Department of Gastroenterology, College of Korean Medicine, Kyung Hee University, Seoul 02447, Republic of Korea.; 4 Department of Longevity and Biofunctional Medicine, Pusan National University School of Korean Medicine, Yangsan 50612, Republic of Korea.

**Keywords:** *Atractylodes macrocephala* Koidzumi, colon, interstitial cells of Cajal, intestinal transit, smooth muscle contraction, electrophysiology

## Abstract

*Atractylodes macrocephala* Koidzumi (AMK) is a traditional herbal medicine used for digestive disorders, yet its effects on colonic motility remain poorly understood. This study aimed to investigate the impact of AMK on human colonic contractility and pacemaker activity of interstitial cells of Cajal (ICCs), as well as its *in vivo* effect on intestinal transit. Human colonic tissues were obtained during non-obstructive colon surgery and used to assess spontaneous smooth muscle contractions and migrating motor complexes (MMCs). Electrophysiological recordings of pacemaker potentials were performed in murine colonic ICCs using whole-cell patch clamp. Pharmacological studies examined the involvement of ATP-sensitive K⁺ channels and cAMP signaling. The intestinal transit rate (ITR) was evaluated in a neostigmine-induced hypermotility mouse model. AMK treatment significantly reduced spontaneous contractions and MMCs in human colonic segments in a dose-dependent manner. In muine colonic ICCs, AMK suppressed pacemaker potentials, with an IC₅₀ of 37.89 µg/mL. This inhibitory effect was reversed by glibenclamide and 8-bromo-cAMP, suggesting involvement of ATP-sensitive K⁺ channels and cAMP-dependent pathways. *In vivo*, AMK attenuated neostigmine-induced increases in ITR. These findings highlight AMK's potential as a modulator of gastrointestinal motility.

## Introduction

The regulation of gastrointestinal (GI) motility involves a finely tuned interplay among enteric neurons, smooth muscle cells, PDGFRα^+^ cells, and interstitial cells of Cajal (ICCs), which serve as pacemakers responsible for initiating peristaltic and segmental movements 1-3. Impairment of this network is associated with various functional bowel disorders, including chronic constipation and intestinal dysmotility syndromes 4,5.

*Atractylodes macrocephala* Koidzumi (AMK), a traditional herbal medicine widely used in East Asia, is known for its anti-inflammatory and GI modulating properties 6-9. Although previous studies have reported its beneficial effects on digestive function, the detailed mechanisms by which AMK influences GI motility remain unclear. In particular, its direct impact on colonic smooth muscle contractility and ICCs pacemaker activity has not been thoroughly investigated. In this study, we examined the effects of AMK on spontaneous contractions and migrating motor complexes (MMCs) in human colonic tissues. Additionally, we assessed its influence on pacemaker potentials in murine colonic ICCs using whole-cell patch-clamp techniques. The potential involvement of ATP-sensitive K⁺ channels and cyclic AMP (cAMP) signaling in AMK-induced responses was also evaluated. Furthermore, the physiological relevance of these findings was confirmed *in vivo* through intestinal transit rate (ITR) analysis in mice. These investigations aim to provide novel insights into the regulatory effects of AMK on colonic motility and to explore its potential as a therapeutic agent for GI motility disorders.

## Materials and Methods

### Ethanolic extraction and HPLC-based component analysis of AMK

AMK extract, obtained from the Korea Plant Extract Bank (Ochang, Chungbuk, Republic of Korea), was prepared using ethanol following established protocols 10. HPLC analysis (JASCO, Easton, MD, USA) identified Eudesma-4(15),7(11)-dien-8-one (EDO) and Atractylenolide III (ATO III), using a gradient elution of 50-100% acetonitrile over 50 minutes, with a 5-minute re-equilibration between runs 10.

### Collection and processing of human colon samples

Human colonic tissues were collected from patients undergoing non-obstructive colon cancer surgery at Seoul National University Hospital, with informed consent and the Institutional Review Board (IRB) approval (H-0603-071-170). Specimens from right hemicolectomy and anterior resection were immediately placed in preoxygenated Krebs-Ringer bicarbonate (KRB) solution (97% O₂, 3% CO₂, pH 7.3-7.4) as previously described 11-13.

### Recording isometric tension in colonic muscle preparations

Smooth muscle contractions were recorded from human colonic strips *in situ* using a modified version of a previously described method 12. Following removal of the mucosa and submucosa, muscle layers were cut into strips (5-6 mm in length × 2-3 mm in width) and mounted on isometric force transducers in aerated (97% O₂, 3% CO₂) KRB solution at 36.5 ± 0.5 °C. After a 60-minute stabilization, tissues were stretched to 1 mN and equilibrated for an additional 60 minutes.

### Measurement of contractile activity in colonic segments

MMCs activity was recorded *in situ* from human colonic segments using a modified method based on previous protocols 13. Longitudinally opened tissue segments (5-6 cm × 2 cm) with intact mucosa and submucosa were prepared, and circular muscle tension was measured at proximal, middle, and distal sites (2 cm apart) using sutures connected to isometric force transducers (Biopac Systems, Inc., Goleta, CA, USA) via micro serrefines (Fine Science Tools, Forster City, CA, USA). After being stretched to 10 mN, tissues were equilibrated for at least 2 hours in continuously perfused, aerated (97% O₂, 3% CO₂) KRB solution at 36.5 ± 0.5 °C.

### Data acquisition and quantitative analysis of human colonic smooth muscle responses

Mechanical responses were digitized using Acknowledge software (Biopac Systems, Inc., Goleta, CA, USA) and analyzed offline with Clampfit (v10.2, Molecular Devices, San Jose, CA, USA). For spontaneous activity, area under the curve (AUC), amplitude, and frequency were evaluated over a 5-minute period, while AUC over 10 minutes was assessed for MMCs. Contractile changes following AMK treatment were expressed as a percentage of control (pre-treatment) values for comparison 12,13.

### Animal experiments

A total of 56 neonatal mice (3-7 days old) and 42 adult male mice (7 weeks old) were obtained from Samtako Bio Korea for ICCs and ITR experiments, respectively. Mice were housed under specific pathogen-free conditions with controlled temperature (20 ± 2 °C), humidity (49 ± 5%), and a 12-hour light/dark cycle, with free access to food and autoclaved water. General health indicators such as fur condition, feeding, defecation, and behavior were monitored daily. For ICCs analysis, cells were observed microscopically after preparation. In ITR studies, AMK was administered orally. All animal procedures were approved by the Pusan National University IACUC (PNU-2023-0315).

### ICCs preparation and electrophysiological analysis

After removal of the colon, luminal contents were flushed with KRB solution, and tissues were pinned in a Sylgard-coated dish for mucosal removal. The trimmed colon was minced and incubated in Hank's solution for 30 minutes, followed by enzymatic dissociation. Isolated cells were plated on collagen-coated coverslips and maintained at 37 °C in smooth muscle growth medium (Clonetics, San Diego, CA, USA) supplemented with stem cell factor (Sigma-Aldrich, St. Louis, MO, USA). Membrane potentials of cultured ICCs were measured using whole-cell patch clamp with an Axopatch 200B amplifier (Axon Instruments, Foster, CA, USA). Data were analyzed using pCLAMP and Origin software (version 2018; MicroCal, Northampton, MA, USA). All recordings were performed at 30-33 °C. ICCs exhibit a characteristic morphology with multiple processes, clearly distinguishable from smooth muscle cells. Following primary culture, cells with typical ICC morphology were identified, and patch-clamp experiments were performed within 12 hours on spontaneously active cluster-type ICCs.

### Analysis of intestinal transit rate (ITR) efficiency

Thirty minutes after oral administration of AMK, Evans Blue was given to assess intestinal transit. Mice were then sacrificed, and the ITR was calculated as the percentage of the total intestinal length traversed by the dye.

### Statistical analysis

Data are presented as mean ± SEM. Normality and homogeneity were verified prior to statistical analysis. One-way ANOVA followed by Bonferroni post hoc tests was performed using Prism 6.0 (GraphPad Software, La Jolla, CA, USA), with p < 0.05 considered statistically significant.

## Results

### Impact of AMK on spontaneous smooth muscle contractions in human colonic tissue

**Figure 1:**
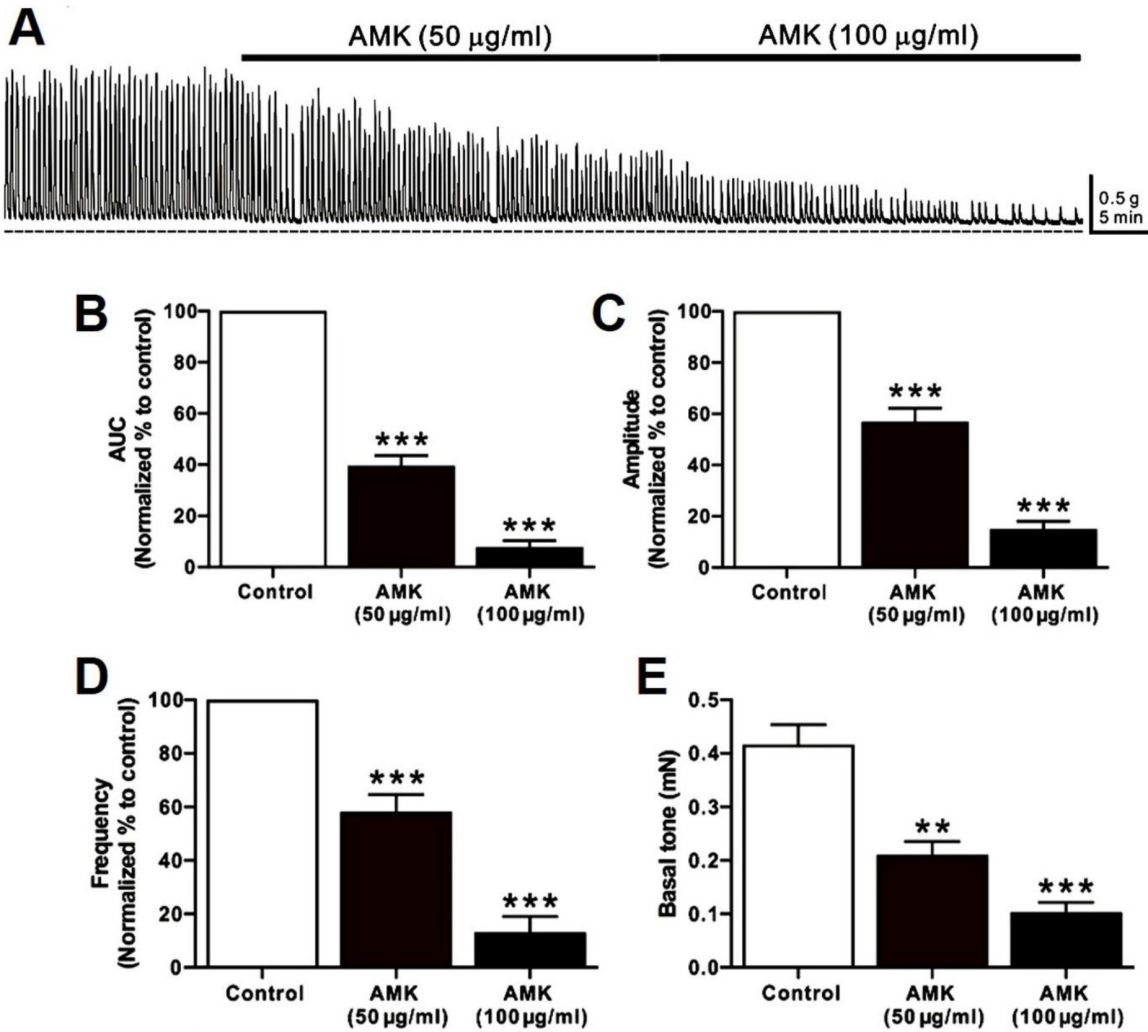
**Impact of AMK on spontaneous contractions in human colonic smooth muscle strips *in situ*. (A)** Representative raw data indicating that AMK enhances spontaneous contractions in a dose-dependent fashion (50-100 µg/mL). Summary graphs depict the influence of AMK on **(B)** AUC, **(C)** amplitude, **(D)** frequency, and **(E)** basal tone. Statistically significant differences are indicated (**p < 0.01, ***p < 0.001) compared to control. AUC: area under the curve. AMK: *Atractylodes macrocephala* Koidzumi.

Spontaneous contractions of human colonic smooth muscle strips were recorded *in situ* (Fig. 1A), and parameters such as AUC, amplitude, frequency, and basal tone were analyzed over a 5-minute period (Table 1). Following AMK treatment, all measured parameters showed a significant, dose-dependent reduction. These results indicate that the overall decline in colonic contractile activity induced by AMK is largely attributable to decreased amplitude and frequency of spontaneous contractions (Fig. 1B-E).

### Influence of AMK on MMCs in human colonic segments

**Figure 2:**
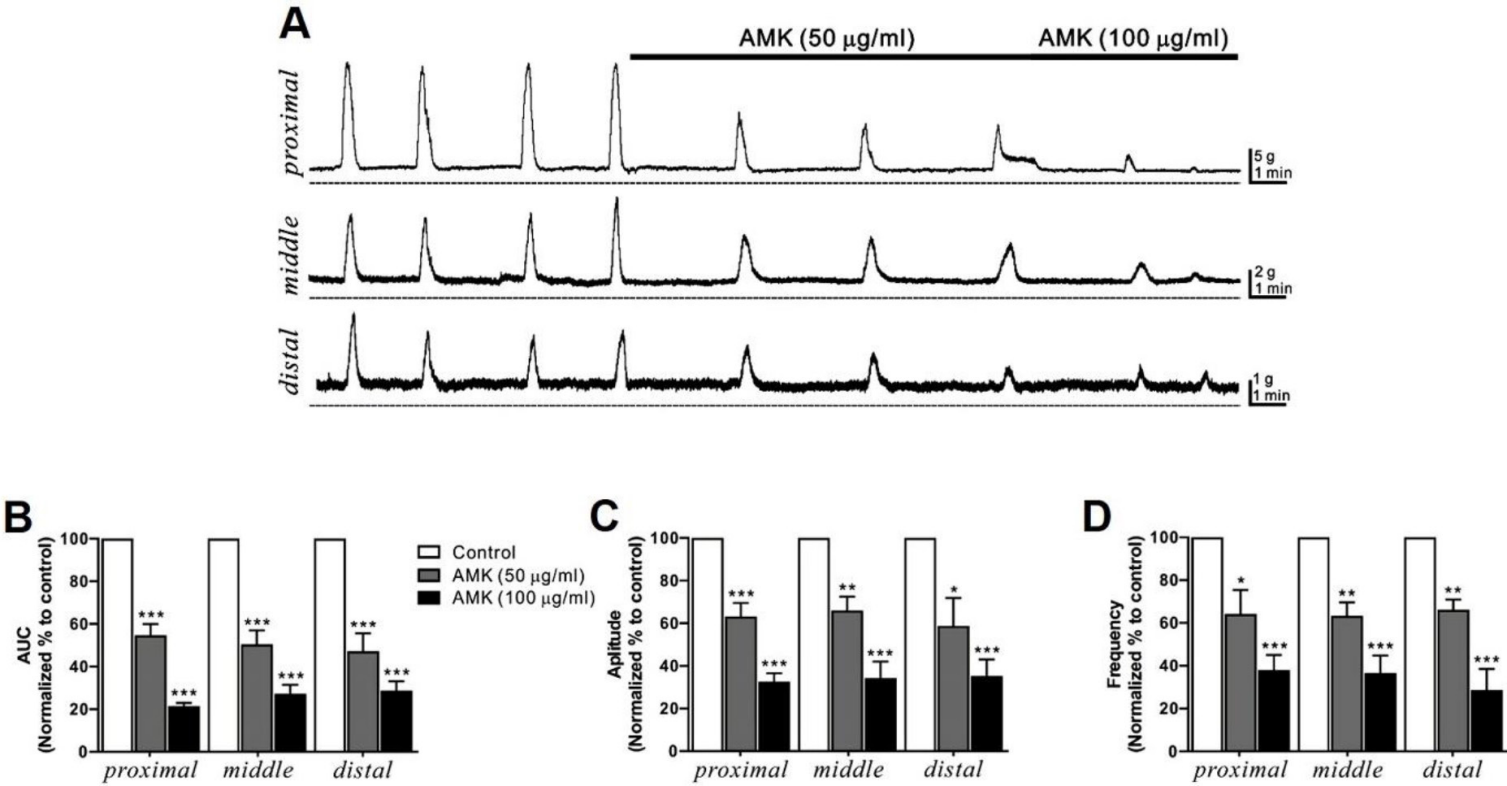
**AMK's influence on MMCs in human colonic segments *in situ*. (A)** Representative raw traces demonstrate that AMK dose-dependently (50-100 µg/mL) enhanced MMCs activity across the proximal, middle, and distal regions of the human colon. Dashed lines represent baseline tension. Summary graphs illustrate the effects of AMK on **(B)** AUC, **(C)** amplitude, and **(D)** frequency at the three colonic sites. Statistical significance: *p < 0.05, **p < 0.01, and ***p < 0.001 versus control. AUC: area under the curve. AMK: *Atractylodes macrocephala* Koidzumi.

MMCs are rhythmic, propulsive contractions of the GI tract driven by neural mechanisms, and are often referred to as “propagating contractile complexes” or simply “peristalsis.” These large-scale contractions facilitate distal movement of luminal contents and play a critical role in digestion and defecation. In this study, MMCs were recorded *in situ* from human colonic segments at three different regions—proximal, middle, and distal (Fig. 2A)—and their AUC, amplitude, and frequency were analyzed over a 10-minute period (Table 2-4). Administration of AMK led to a dose-dependent reduction in all measured parameters across all three sites (Fig. 2B-D). These findings suggest that AMK may have a broad inhibitory effect on colonic motility of the human colon.

### Effect of AMK on spontaneous pacemaker activity in mouse colonic ICCs

**Figure 3:**
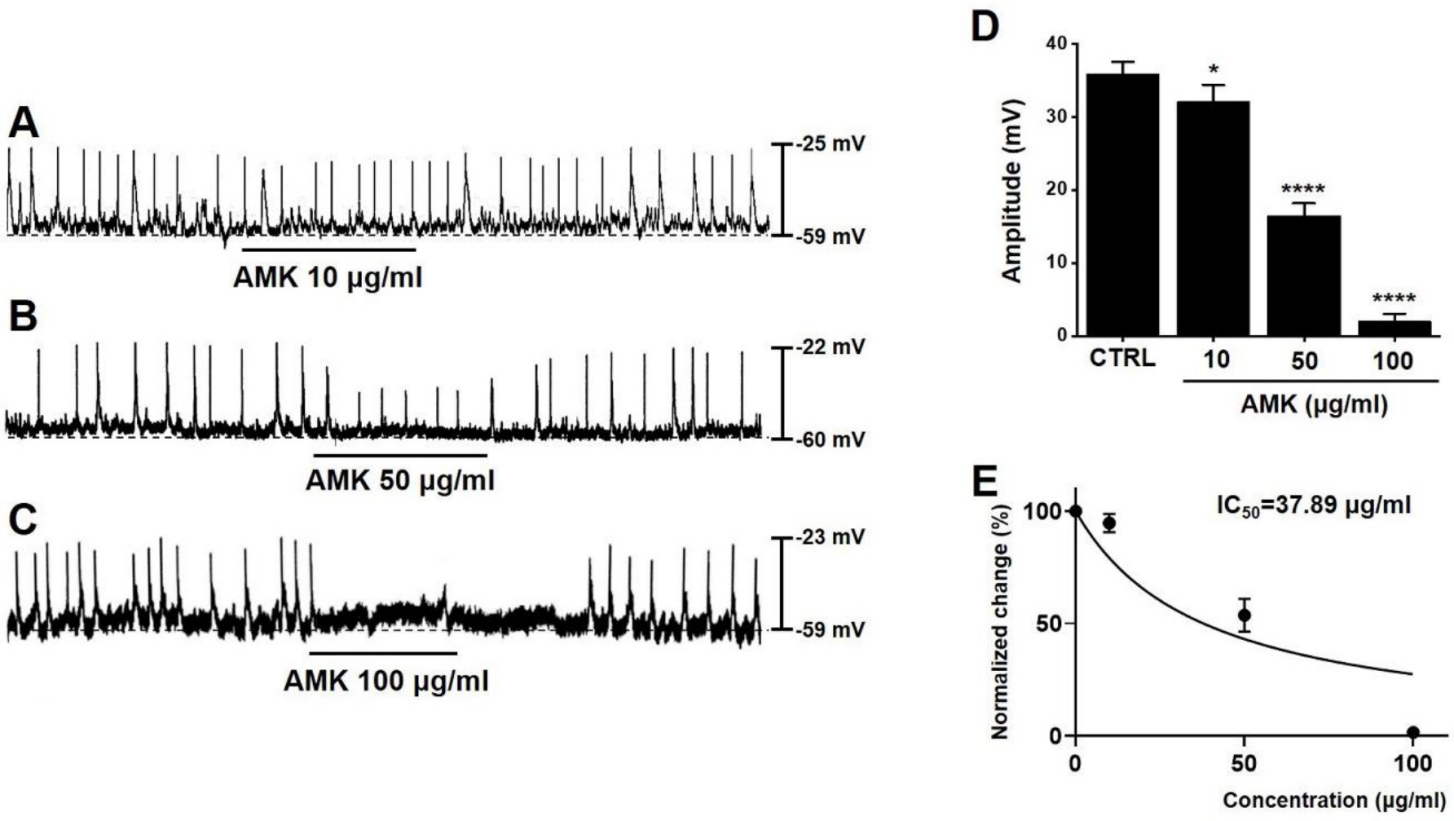
**Impact of AMK on pacemaker potentials in mouse colonic ICCs. (A-C)** AMK suppressed pacemaker potentials in a concentration-dependent manner. **(D)** Summary graph displaying the extent of inhibition in amplitude caused by AMK treatment. **(E)** The half-maximal inhibitory concentration (IC₅₀) of AMK for amplitude reduction was calculated to be 37.89 µg/mL. Statistical significance: *p < 0.05, ****p < 0.0001. CTRL: control. AMK: *Atractylodes macrocephala* Koidzumi.

To evaluate the influence of AMK on murine colonic ICCs, we conducted electrophysiological experiments using current-clamp recordings. Spontaneous pacemaker potentials were observed in murine colonic ICCs, with an average amplitude of 35.85 ± 1.70 mV (n = 48) (Fig. 3A-C). Treatment with AMK extract resulted in a dose-dependent suppression of these potentials, with measured amplitudes of 32.05 ± 2.32 mV at 10 μg/mL (*p < 0.05), 16.48 ± 1.68 mV at 50 μg/mL (****p < 0.0001), and 2.05 ± 1.11 mV at 100 μg/mL (****p < 0.0001) (Fig. 3D). Furthermore, the half-maximal inhibitory concentration (IC_50_) of AMK on the amplitude of pacemaker potentials was determined to be 37.89 µg/mL (Fig. 3E). These results indicate that AMK significantly suppresses pacemaker potential generation in colonic ICCs.

### Role of ATP-sensitive K⁺ channels in AMK-mediated modulation of pacemaker activity in mouse colonic ICCs

**Figure 4:**
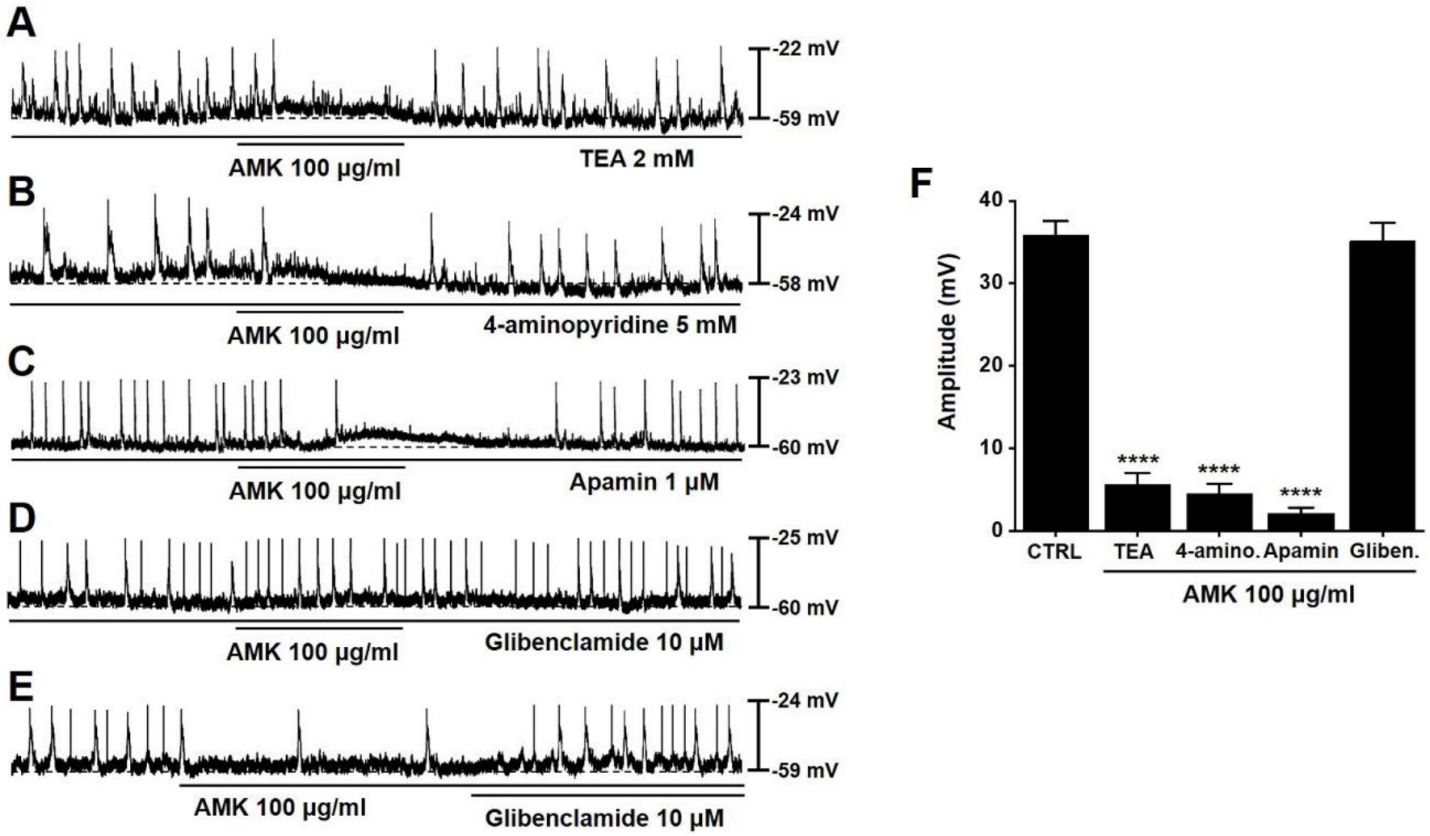
** Influence of different K⁺ channel blockers on AMK-induced inhibition of pacemaker potentials in mouse colonic ICCs. (A)** TEA pretreatment showed no effect on AMK-induced inhibition. **(B)** Similarly, 4-aminopyridine did not alter the inhibitory response. **(C)** Apamin also failed to modify the suppression of pacemaker activity by AMK. **(D)** In contrast, glibenclamide effectively prevented the AMK-induced inhibition. **(E)** The reduction in pacemaker potential caused by AMK was reversed following glibenclamide treatment. **(F)** Summary graph showing the amplitude of inhibition under each pretreatment condition. Statistical significance: ****p < 0.0001. CTRL: control. AMK: *Atractylodes macrocephala* Koidzumi. TEA: tetraethylammonium. 4-amino: 4-aminopyridine. Gliben: glibenclamide.

To examine whether K⁺ channels are involved in the AMK-induced modulation of pacemaker activity, we applied various K⁺ channel blockers. The inhibitory effect of AMK on pacemaker potentials persisted in the presence of tetraethylammonium (TEA), as well as with 4-aminopyridine and apamin (Fig. 4A-C). However, glibenclamide alone did not alter pacemaker activity, and under co-treatment conditions, AMK failed to exert its inhibitory effect (Fig. 4D). Furthermore, glibenclamide effectively reversed the suppression induced by AMK (Fig. 4E). When 100 μg/mL AMK was applied, the average amplitudes of pacemaker potentials were significantly reduced: 5.63 ± 1.37 mV in TEA, 4.53 ± 1.20 mV in 4-aminopyridine, and 2.10 ± 0.71 mV in apamin-treated cells (all ****p < 0.0001) (Fig. 4F). These findings indicate that the inhibitory action of AMK on pacemaker potential generation is likely mediated via ATP-sensitive K⁺ channels in colonic ICCs.

### Role of cAMP signaling in AMK-mediated modulation of pacemaker activity in mouse colonic ICCs

**Fig 5:**
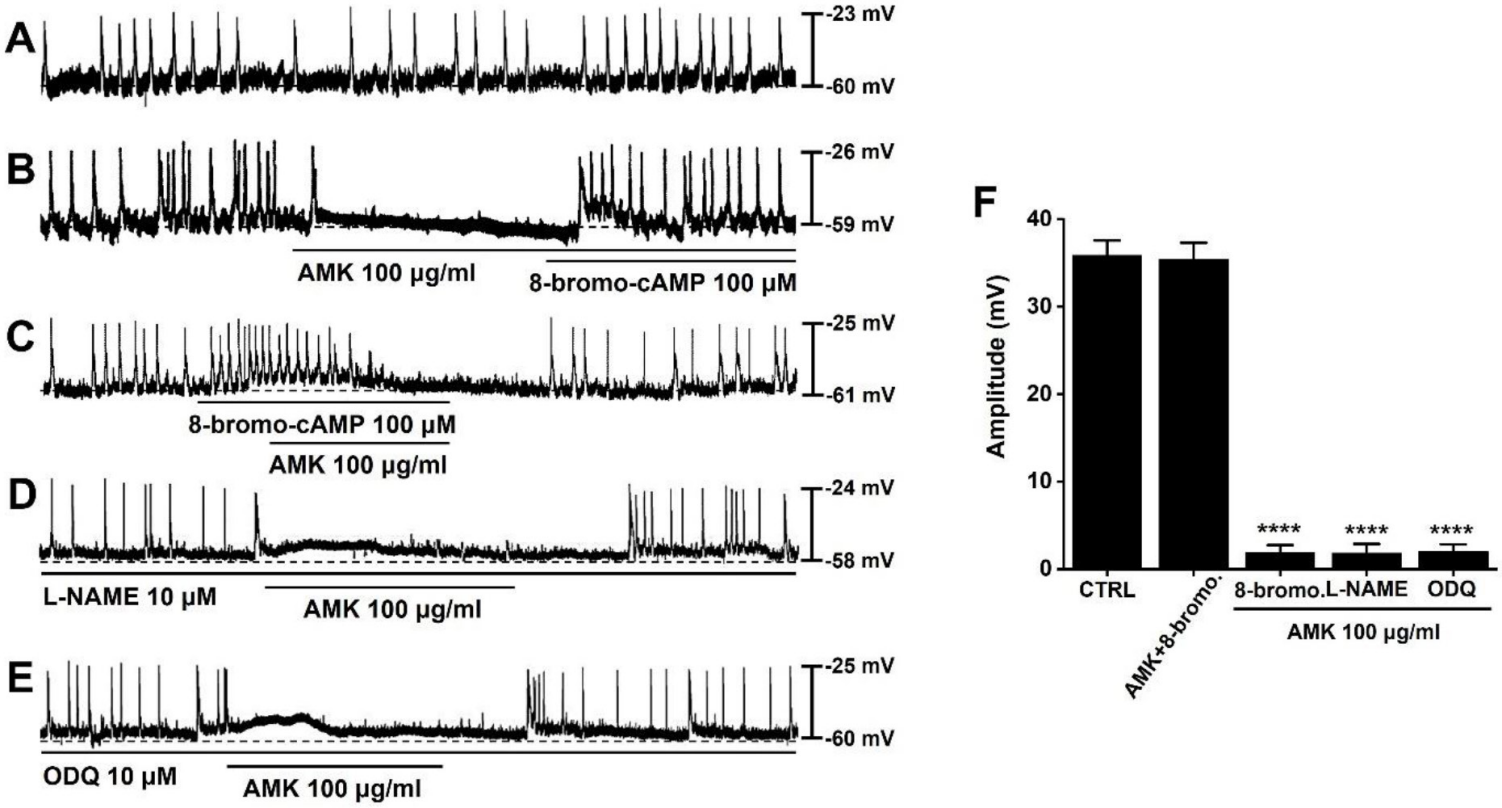
** Modulatory effects of 8-bromo-cAMP, L-NAME, and ODQ on AMK-induced suppression of pacemaker potentials in mouse colonic ICCs. (A)** Spontaneous pacemaker potentials could be observed in mouse colonic ICCs. **(B)** Application of 8-bromo-cAMP reversed the inhibition of pacemaker potentials by AMK. **(C)** 8-bromo-cAMP elevated the frequency of pacemaker potentials; under these conditions, AMK continued to suppress the activity. **(D)** Pretreatment with L-NAME had no observable impact on AMK-induced inhibition. **(E)** Similarly, ODQ did not alter the suppressive effect of AMK on pacemaker potentials. **(F)** Summary graph depicting the degree of amplitude inhibition across the various treatment combinations. Statistical significance: ****p < 0.0001. CTRL: control. AMK: *Atractylodes macrocephala* Koidzumi. 8-bromo: 8-bromo-cAMP. L-NAME: NG-nitro-L-arginine methyl ester. ODQ: 1H-[1,2,4]oxadiazolo[4,3-a]quinoxalin-1-one.

To explore the potential role of cAMP in the regulation of pacemaker activity, we applied 8-bromo-cAMP, a membrane-permeable analog of cAMP. Spontaneous pacemaker potentials could be observed in mouse colonic ICCs (Fig. 5A). Under conditions where pacemaker activity was suppressed by AMK, administration of 8-bromo-cAMP (100 μM) restored and enhanced the generation of pacemaker potentials (Fig. 5B). Furthermore, under conditions where 8-bromo-cAMP (100 μM) elevated the frequency of pacemaker potentials and caused a slight membrane depolarization, AMK was found to suppress the generation of these pacemaker potentials (Fig. 5C). To further assess whether the nitric oxide (NO) signaling pathway contributes to AMK-induced effects, we co-applied AMK with L-NAME (a nitric oxide synthase inhibitor) and ODQ (a guanylate cyclase inhibitor). Neither L-NAME (10 μM) nor ODQ (10 μM) significantly altered the AMK-mediated inhibition of pacemaker activity (Fig. 5D and E). The mean amplitudes of pacemaker potentials recorded under each condition were as follows: 35.43 ± 1.90 mV in AMK+8-bromo-cAMP, 1.88 ± 0.98 mV (****p < 0.0001) in 8-bromo-cAMP, 1.83 ± 1.10 mV (****p < 0.0001) in L-NAME, and 2.10 ± 0.95 mV (****p < 0.0001) in ODQ (Fig. 5F). These data support that AMK's suppressive effect on pacemaker potential generation is mediated primarily through cAMP-dependent pathways rather than through NO signaling in colonic ICCs.

### AMK suppresses neostigmine-induced increase in ITR

Given the *in vitro* findings showing that AMK suppresses pacemaker activity in colonic ICCs, we further investigated its effects on ITR in an *in vivo* model. Neostigmine is known to enhance intestinal motility, increase colonic contractions, and elevate ITR. In mice treated with neostigmine, oral administration of AMK at doses of 0.5 g/kg and 1 g/kg significantly attenuated the neostigmine-induced elevation in ITR (Fig. 4). These results imply that AMK-induced reduction of ITR might be attributed to its inhibitory effect on the pacemaker activity of ICCs in the colon.

## Discussion

This study provides novel evidence that AMK, a traditional herbal medicine, exerts a suppressive effect on colonic motility by modulating both smooth muscle contractility (Fig. 1 and 2) and the pacemaker activity of ICCs (Fig. 3,4, and 5). Using a combination of *ex vivo* human colonic tissues, electrophysiological analysis in murine colonic ICCs, and an *in vivo* intestinal transit model, we demonstrated that AMK significantly inhibits GI motor activity through mechanisms involving ATP-sensitive K⁺ channels and cAMP-dependent signaling pathways.

A particularly significant aspect of this study lies in its use of human colonic tissues to evaluate the direct impact of AMK on spontaneous smooth muscle contractions and MMCs. Conducting GI motility researches using human specimens poses a considerable challenge due to the limited availability of viable, ethically sourced tissue samples. Most prior studies in this field have relied on animal models or immortalized cell lines, which may not fully recapitulate the complexity of human colonic motility. The fact that our experimental data were obtained from freshly isolated, non-obstructed human colon segments enhances the translational relevance and clinical applicability of our findings. This approach adds substantial value to the study by providing a rare, physiologically accurate model to explore the effects of AMK in human GI tissues. In these human tissue preparations, AMK reduced the amplitude, frequency, and AUC of spontaneous contractions (Fig. 1) and MMCs (Fig. 2) in a concentration-dependent manner, indicating broad inhibition of colonic motor activity. Given that MMCs are essential for peristalsis and colonic clearance, AMK's attenuation of this rhythmic activity supports its potential as a treatment option for disorders involving hypermotility or spasmodic bowel movement.

Electrophysiological studies further revealed that AMK potently suppressed spontaneous pacemaker potentials in murine colonic ICCs, with an IC₅₀ of 37.89 µg/mL (Fig. 3). ICCs are known to coordinate smooth muscle contractions by generating rhythmic electrical activity 14-16. Thus, AMK-induced inhibition of ICC function may underlie its broader suppressive effect on colonic motility. The mechanistic basis of this inhibition was explored using pharmacological blockers. Among several tested K⁺ channel inhibitors, only glibenclamide—an ATP-sensitive K⁺ channel blocker 17—reversed the inhibitory effect of AMK, suggesting that AMK mediates its action through ATP-sensitive K⁺ channels. The pacemaker activity of ICCs is known to be regulated by a complex interplay of multiple ion channels. These include non-selective cation channels 18, calcium-activated chloride channels 19, various potassium channels 20, as well as voltage-dependent calcium channels 21. The coordinated activity of these ion channels generates the rhythmic depolarizations that underlie GI motility. Therefore, modulation of any of these channels can significantly impact ICCs excitability and, consequently, gut motility. In this study, it was shown that AMK modulated ICCs activity through ATP-sensitive K⁺ channels. Additionally, restoration of pacemaker potentials by 8-bromo-cAMP implicates the involvement of a cAMP-dependent mechanism. Interestingly, inhibitors of nitric oxide signaling (L-NAME 22 and ODQ 23) did not affect AMK-induced responses, indicating that AMK's action is independent of NO-cGMP pathways. In addition to the mechanistic findings, this study underscores the potential of AMK as a complementary therapeutic option for GI disorders 24. GI diseases, including both structural and functional abnormalities, encompass a wide range of symptoms, from mild discomfort to severe, life-altering conditions 25. Among these, functional gastrointestinal disorders (FGIDs), such as irritable bowel syndrome (IBS) and functional constipation, often lack clear organic pathology, making them difficult to manage using conventional Western medical approaches alone 26.

Traditional Chinese medicine, including the use of herbal remedies like AMK, offers a holistic and individualized strategy that may minimize the side effects associated with pharmacological interventions and improve patient quality of life 27,28. Especially in FGIDs, where psychosomatic and neuroenteric factors play crucial roles, herbal treatments can serve as adjunctive or alternative options that target not only the symptoms but also the underlying dysregulation of GI motility 26,29,30. Our findings that AMK modulates smooth muscle contractility and ICC pacemaker activity support its potential application in clinical settings where excessive or uncoordinated GI motility is a concern. Furthermore, by integrating pharmacological validation with physiologically relevant models, including human tissues and *in vivo* assays, our study provides a robust translational foundation for the future development of AMK-based therapeutics for GI motility disorders.

**Figure 6:**
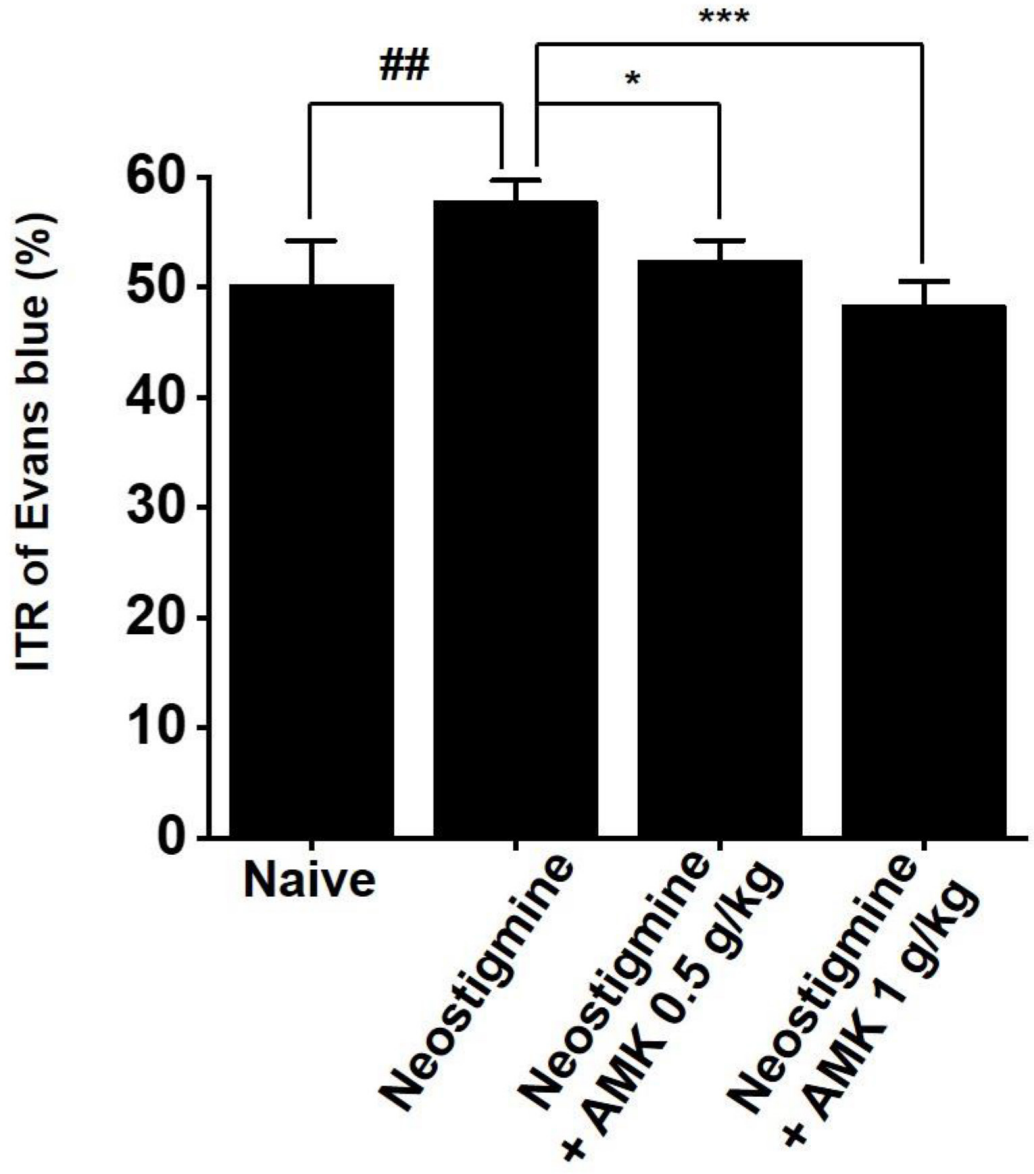
**Impact of AMK on ITR in neostigmine-treated mice *in vivo*.** Administration of AMK at doses of 0.5 g/kg and 1 g/kg significantly reduced the neostigmine-induced enhancement of ITR. Statistical significance: ## p < 0.01, *p < 0.05, and ***p < 0.01. AMK: *Atractylodes macrocephala* Koidzumi. ITR: Intestinal Transit Rate.

**Figure 7:**
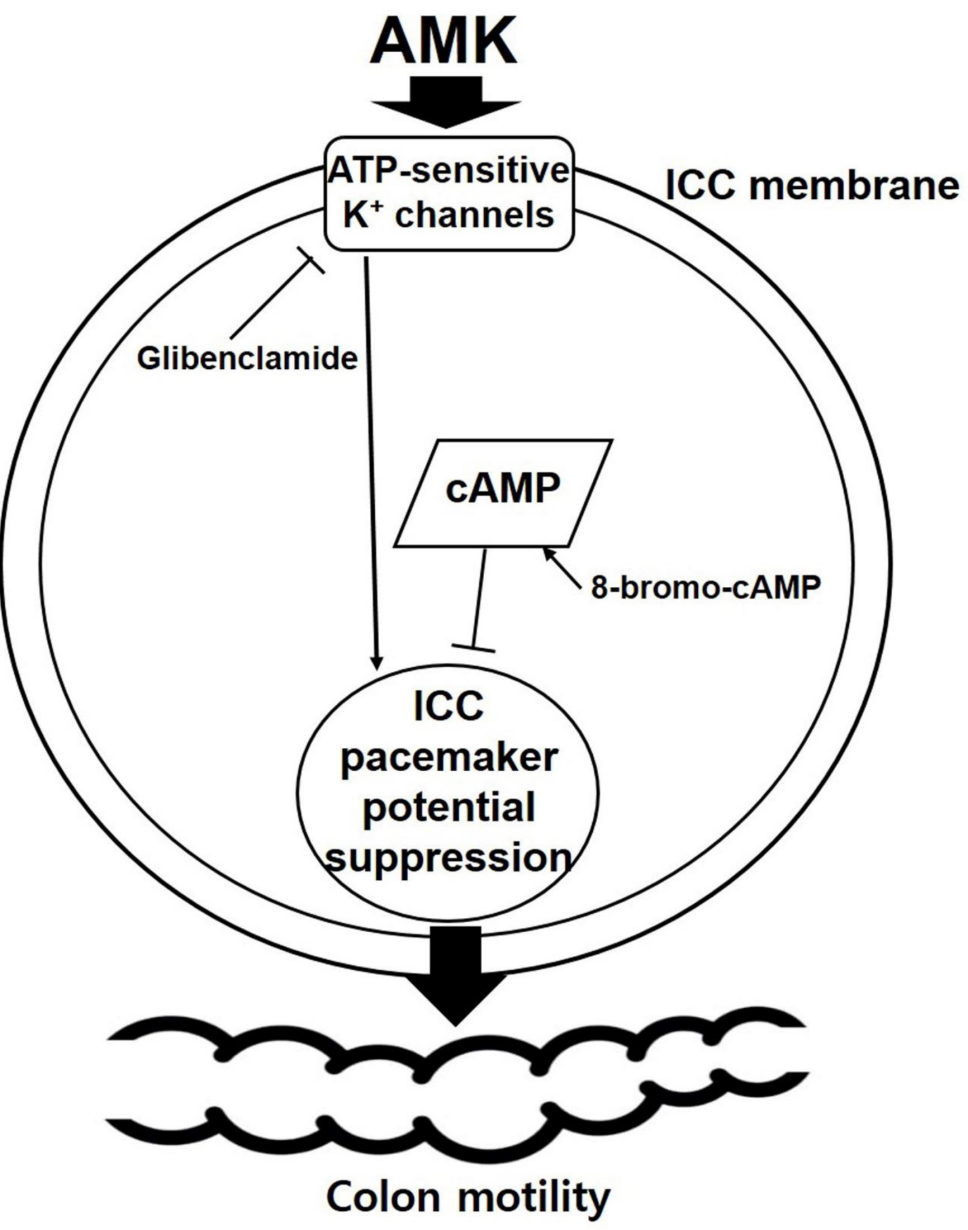
Proposed mechanism of AMK action on colonic ICCs. AMK inhibits the pacemaker activity of colonic ICCs in a dose-dependent manner (IC₅₀ = 37.89 µg/mL). This inhibitory effect is reversed by glibenclamide, an ATP-sensitive K⁺ channel blocker, and 8-bromo-cAMP, a membrane-permeable cAMP analog, suggesting that AMK modulates pacemaker activity via ATP-sensitive K⁺ channels and cAMP-dependent signaling pathways. The suppression of ICC pacemaker potentials may contribute to reduced colonic motility. AMK: *Atractylodes macrocephala* Koidzumi.

The *in vivo* component of this study corroborated the *ex vivo* and *in vitro* findings. AMK significantly suppressed neostigmine-induced elevations in ITR, reinforcing the hypothesis that AMK attenuates motility by downregulating pacemaker activity (Fig. 6). This functional outcome is consistent with the observed electrophysiological and mechanical data, and highlights the translational relevance of our results. Following AMK administration, we monitored body weight, food and water intake, and general activity levels in mice. No significant behavioral abnormalities or adverse effects were observed. Additionally, potential toxicity in non-GI organs such as the liver and kidneys was indirectly assessed through gross anatomical examination, which revealed no notable abnormalities. The dosage range used in this ITR study (0.5-1 g/kg) was not based on specific prior studies of AMK, but reflects commonly accepted ranges for herbal or natural products used in animal experiments, and is generally considered to be within a safe range 31-33. However, we acknowledge that long-term toxicity and potential immunological effects of repeated AMK administration should be further investigated in future studies. The observed inhibitory effects of AMK on human colonic contractility and on ITR in the mouse model are likely mediated through a shared mechanism involving the suppression of pacemaker activity in ICCs. ICCs play a central role in generating and coordinating rhythmic contractions in the GI tract. In our study, AMK significantly suppressed pacemaker potentials in colonic ICCs, which would lead to decreased excitation of surrounding smooth muscle and, consequently, reduced motility. This mechanistic inhibition at the cellular level provides a plausible explanation for both the dampening of spontaneous and MMC-like contractions in human colon tissue and the delayed transit observed *in vivo* in the neostigmine-induced hypermotility model. Therefore, the findings from both the *ex vivo* and *in vivo* systems are consistent and point toward a unified inhibitory mechanism of AMK on GI motility through ICC modulation (Fig. 7).

Previous studies have reported the GI-modulating properties of AMK, attributing its effects to components such as EDO and ATO III 10. EDO and ATO III are known as major components of AMK and were used for component analysis in this study 34. Although there have been no studies investigating the effects of EDO on ICCs, ATO III has been reported to modulate ICCs indirectly by increasing c-kit and stem cell factor expression in intestinal tissue, albeit not through electrophysiological approaches 35. However, the possibility of ICCs modulation via ion channel regulation by these compounds has not been explored. In addition, to our knowledge, this is the first study to provide electrophysiological evidence for AMK's direct action on ICCs and its downstream impact on colonic motility. The identification of ATP-sensitive K⁺ channels and cAMP signaling as key mediators offers mechanistic insight into how AMK may exert its regulatory effects, and sets the foundation for further investigation into its therapeutic applications in GI motility disorders.

Despite these significant findings, this study has several limitations. First, while human tissues were used for contractility assays, ICCs recordings were performed on murine colonic cells because it is technically challenging to study ICCs using electrophysiological methods in human tissues. Species-specific differences may influence ICC responsiveness to herbal compounds. Second, although AMK contains multiple active constituents, we did not isolate individual compounds in this study; thus, the specific molecular targets remain to be elucidated. Future studies using purified components and knockout animal models will help clarify the precise signaling pathways involved.

IBS and other functional GI disorders are often associated with abnormal gut motility, particularly hypermotility in patients with diarrhea-predominant IBS, which contributes to symptoms such as abdominal pain and urgency. In this study, AMK was shown to suppress excessive spontaneous contractions and MMCs in human colonic tissues, reduce pacemaker activity in ICCs electrophysiologically, and normalize intestinal transit in a neostigmine-induced hypermotility mouse model. These findings provide physiological evidence that AMK may help regulate hypermotility-related symptoms. Thus, AMK may serve as a potential therapeutic modulator for disorders characterized by stress-related or diarrhea-type motility abnormalities. Importantly, these results suggest that AMK has clinically relevant potential as a natural therapeutic agent for managing functional bowel disorders associated with dysmotility. Further studies using validated IBS animal models—including assessments of visceral pain behavior, inflammatory markers, and gut microbiota composition—will be essential to support the clinical applicability of AMK.

In conclusion, our results demonstrate that AMK exerts inhibitory effects on colonic motility by suppressing pacemaker activity in ICCs, involving ATP-sensitive K⁺ channels and cAMP-dependent mechanisms. These findings suggest that AMK may have therapeutic potential as a modulator of GI motility and offer new perspectives for its application in the management of functional bowel disorders.

## Figures and Tables

**Table 1:** Relative values of AUC, amplitude, frequency, and basal tone for spontaneous contractions after treatment with each dose of AMK in human colonic smooth muscle strips *in situ*.

Segment	Parameter (n)	Dose of AMK (μg/ml)	
		50	100
Colon	AUC (9)	39.5 ± 8.9 ***	7.8 ± 5.8 ***
	Amplitude (8)	56.9 ± 11.9 ***	14.9 ± 6.9 ***
	Frequency (8)	58.2 ± 14.5 ***	13.1 ± 13.2 ***
	Basal tone (7)	0.21 ± 0.05 **	0.10 ± 0.04 ***

Data are expressed as the mean ± SEM. ** p < 0.01, and *** p < 0.001 vs. each control.

**Table 2:** Relative AUC values (% of control) for MMCs after treatment with each dose of AMK in human colonic segments *in situ*.

Segment	Site	Dose of AMK (μg/ml)	
		50	100
Colon	Proximal	54.7 ± 5.2 ***	21.5 ± 1.5 ***
	Middle	50.4 ± 6.6 ***	27.3 ± 4.2 ***
	Distal	47.2 ± 8.5 ***	28.7 ± 4.4 ***

Data are expressed as the mean ± SEM. *** p < 0.001 vs. each control.

**Table 3:** Relative amplitude values (% of control) for MMCs after treatment with each dose of AMK in human colonic segments *in situ*.

Segment	Site	Dose of AMK (μg/ml)	
		50	100
Colon	Proximal	63.2 ± 6.3 ***	32.6 ± 3.9 ***
	Middle	66.1 ± 6.4 **	34.3 ± 7.7 ***
	Distal	58.7 ± 13.1 *	35.3 ± 7.8 ***

Data are expressed as the mean ± SEM. * p < 0.05, ** p < 0.01, and *** p < 0.001 vs. each control.

**Table 4:** Relative frequency values (% of control) for MMCs after treatment with each dose of AMK in human colonic segments *in situ*.

Segment	Site	Dose of AMK (μg/ml)	
		50	100
Colon	Proximal	64.2 ± 11.2 *	38.0 ± 7.1 ***
	Middle	63.4 ± 6.2 **	36.6 ± 8.2 ***
	Distal	66.2 ± 4.8 **	28.6 ± 9.9 ***

Data are expressed as the mean ± SEM. * p < 0.05, ** p < 0.01, and *** p < 0.001 vs. each control.
